# A Multicenter Study of Acute Abdomen in Children With Acute Lymphoblastic Leukemia: CCCG‐ALL‐2015

**DOI:** 10.1002/cam4.71090

**Published:** 2025-08-21

**Authors:** Wenting Gan, Weina Zhang, Jiaoyang Cai, Xiaofan Zhu, Jie Yu, Peifang Xiao, Ju Gao, Yongjun Fang, Changda Liang, Xue Li, Fen Zhou, Xiaowen Zhai, Xiaoxiao Xu, Xin Tian, Aiguo Liu, Ningling Wang, Jiashi Zhu, Frankie Wai‐Tsoi Cheng, Liangchun Yang, Ge Zhang, Shuhong Shen, Hua Jiang, Lingzhen Wang

**Affiliations:** ^1^ Department of Hematology/Oncology Guangzhou Medical University Affiliated Women and Children's Medical Center Guangzhou China; ^2^ Department of Hematology/Oncology Shanghai Children's Medical Center, Shanghai Jiao Tong University of School of Medicine Shanghai China; ^3^ State Key Laboratory of Experimental Hematology and Division of Pediatric Blood Diseases Center Institute of Hematology and Blood Diseases Hospital, Chinese Academy of Medical Sciences and Peking Union Medical College Tianjin China; ^4^ Department of Hematology/Oncology Chongqing Medical University Affiliated Children's Hospital Chongqing China; ^5^ Department of Hematology/Oncology Children's Hospital of Soochow University Suzhou China; ^6^ Department of Pediatrics West China Second University Hospital, Sichuan University, Key Laboratory of Birth Defects and Related Disease of Women and Children, Ministry of Education Chengdu China; ^7^ Department of Hematology/Oncology Children's Hospital of Nanjing Medical University Nanjing China; ^8^ Department of Hematology/Oncology Jiangxi Provincial Children's Hospital Nanchang China; ^9^ Department of Pediatrics Qilu Hospital of Shandong University Jinan China; ^10^ Department of Pediatrics Union Hospital of Tongji Medical College, Huazhong University of Science and Technology Wuhan China; ^11^ Department of Hematology/Oncology Children's Hospital of Fudan University Shanghai China; ^12^ Department of Pediatrics Nan Fang Hospital, Southern Medical University Guangzhou China; ^13^ Department of Hematology/Oncology KunMing Children's Hospital Kunming China; ^14^ Department of Pediatrics Tongji Hospital of Tongji Medical College, Huazhong University of Science and Technology Wuhan China; ^15^ Department of Pediatrics Anhui Medical University Second Affiliated Hospital Anhui China; ^16^ Department of Hematology/Oncology Children's Hospital Affiliated to Shanghai Jiao Tong University Shanghai China; ^17^ Department of Pediatrics Hong Kong Children's Hospital, The Chinese University of Hong Kong Hong Kong SAR China; ^18^ Department of Pediatrics Xiangya Hospital Central South University Changsha China; ^19^ Department of Hematology/Oncology Xi'an Northwest Women's and Children's Hospital Xi'an China; ^20^ Department of Pediatrics Affiliated Hospital of Qingdao University Qingdao China

**Keywords:** acute abdomen, acute lymphoblastic leukemia, children, risk factors

## Abstract

**Objective:**

Acute abdomen presents a significant challenge in the treatment of childhood acute lymphoblastic leukemia (ALL), potentially leading to treatment failure and treatment‐related mortality. Results from the multicenter study of CCCG‐ALL‐2015 showed significant improvements in overall survival (OS) and event‐free survival (EFS) for childhood ALL. However, the primary aim of this study was to determine the incidence, risk factors, and clinical impact of acute abdomen in the CCCG‐ALL‐2015 protocol, with the ultimate goal in the future of establishing evidence‐based preventive strategies to reduce its occurrence.

**Methods:**

Patients participating in the CCCG‐ALL‐2015 protocol from January 1, 2015, to December 31, 2019, were included in this analysis. The occurrence of acute abdomen in 7640 patients with ALL was analyzed retrospectively. The data was collected in August 2023 from the Data Center. Acute abdomen analyzed in this study included: acute pancreatitis, acute appendicitis, ileus, enterobrosis, enterorrhagia, peritonitis, enteritis, and others (urinary calculus, anaphylactoid purpura, cholecystitis). The clinical data of patients with acute abdomen were extracted from the database and analyzed.

**Results:**

A total of 7640 patients were recruited in the study, and 512 (6.7%) patients diagnosed with acute abdomen were identified. Of the 512 patients, 50 patients experienced two episodes of acute abdomen, 5 patients had three episodes, and 2 patients had four episodes. Among different types of acute abdomen, the incidence of acute pancreatitis was the highest (4.0%), followed by ileus (1.2%) and acute appendicitis (0.7%). There was no difference in the incidence of acute abdomen between males and females (6.6% vs. 6.9%, *p* = 0.64). The incidence of acute abdomen was associated with age, and patients older than 10 years had a significantly higher incidence rate than those less than 1 year and 1–10 years old (13.0% vs. 5.7%, *p* < 0.0001). The incidence rate of enterorrhagia was significantly higher in patients less than 1 year and older than 10 years than that in patients aged 1–10 years old (1.4% vs. 0.4%, *p* < 0.0001). The incidence of acute abdomen in the intermediate or high‐risk (I/HR) group was higher than that in the low‐risk (LR) group (9.9% vs. 3.7%, *p* < 0.001). Acute abdomen mainly occurred in the induction remission phase (57.3%) and the continuation and reinduction phase (39.3%). A total of 16 patients died from acute abdomen, including four patients in the LR group and 12 patients in the I/HR group. Among the different types of acute abdomen, the mortality rate of enterorrhagia was the highest (16.7%), followed by enterobrosis (9.1%).

**Conclusion:**

This multicenter study investigates the frequency, risk factors, and impact of acute abdomen in the CCCG‐ALL‐2015 protocol, the largest pediatric ALL study in China. We identified acute abdomen in 6.7% of patients, with the highest incidence during the induction remission phase and in older children and those with intermediate or high‐risk ALL. Acute pancreatitis was the most common type, while enterorrhagia had the highest mortality. These findings underscore the need for heightened vigilance and proactive management of acute abdomen to improve outcomes in children with ALL.

**Trial Registration:**

Chinese Clinical Trial Registry: ChiCTR‐IPR‐14005706

## 1 Introduction

Acute lymphoblastic leukemia (ALL) is the most common pediatric hematological malignancy. With advancements in medical technology, refinement of chemotherapy regimens, and the increasing application of diverse targeted therapies, the complete remission and long‐term survival rates for pediatric acute lymphoblastic leukemia patients have significantly improved [1, 2]. However, chemotherapy‐related complications still contribute considerably to treatment‐related mortality. Among these, acute abdomen is a common chemotherapy‐related complication. During chemotherapy, particularly during bone marrow suppression following high‐intensity chemotherapy, acute abdomen often presents with atypical clinical symptoms and signs, posing significant challenges for early diagnosis and treatment [3]. In China, there is limited literature on chemotherapy‐related acute abdomen, and currently no comprehensive childhood data analysis of acute abdomen following chemotherapy, nor established clinical guidelines for its early diagnosis and treatment. In January 2015, the Chinese Children's Cancer Research Group (CCCG) launched the CCCG‐ALL‐2015 multicenter study [4]. By December 31, 2019, 7640 children with ALL enrolled, and their OS and EFS were comparable to those children with ALL in developed countries [5]. This study aims to analyze acute abdomen cases reported in each research center to examine their frequency of occurrence, impact on chemotherapy, and associated risk factors.

## 2 Methods

### 2.1 Study Subjects

From January 1, 2015, to December 31, 2019, eligible children diagnosed with ALL were enrolled in the CCCG‐ALL‐2015 study at each study center. The patients were included in this study with written informed consent from their legal guardians and were under 18 years old at the time of diagnosis.

### 2.2 Treatment Protocols

The whole chemotherapy phase of this treatment protocol [4] is divided into induction remission (weeks 1–7), consolidation (weeks 8–15), continuation therapy and reinduction (weeks 16–34), and maintenance phase (weeks 35–125). The enrolled patients received stratified chemotherapy according to the risk stratification (LR or I/HR).

### 2.3 Data Collection

Patients with acute abdomen were screened from the data registration system and were further confirmed by reviewing the detailed clinical data. The types of acute abdomen analyzed in this study, based on the classification standard of acute abdomen [6] and the collected data, included acute pancreatitis, acute appendicitis, ileus, enterobrosis, enterorrhagia, peritonitis, enteritis, and others (urinary calculus, anaphylactoid purpura, cholecystitis). The information collected included: gender, age, peripheral white blood cell count, immunophenotype, risk stratification, type of acute abdomen, time of onset of acute abdomen, treatment method (either active medical or surgical treatment), treatment outcome and its effect on anti‐leukemia chemotherapy, etc.

### 2.4 Statistics

Data were analyzed using GraphPad Prism 9 statistical software. Categorical data were compared with a chi‐squared test. Gender, age, WBC, immunophenotype, Ph‐ALL, and risk group were tested for associations with the incidence of acute abdomen. A univariate analysis was performed for risk factors leading to death due to acute abdomen, and a binary logistic regression analysis was then performed for those with significance. Fisher's exact test was performed to compare different risk groups. *p* value < 0.05 would be considered statistically significant.

## 3 Results

### 3.1 Type of Acute Abdomen

**FIGURE 1:**
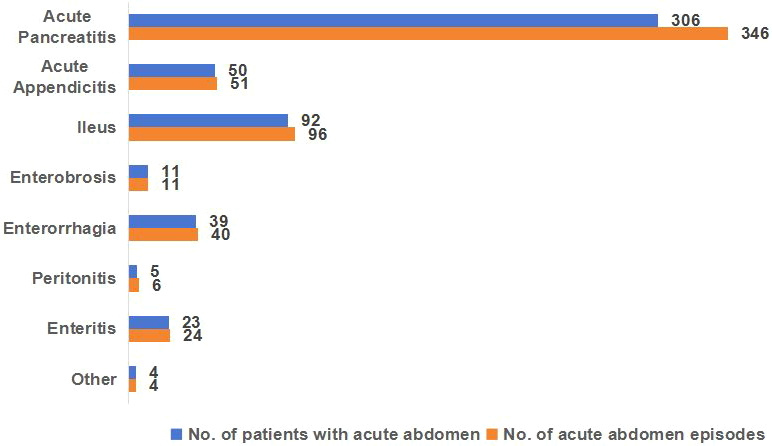
Number and incidence of different types of acute abdomen.

Among the 7640 patients, 512 (6.7%) experienced acute abdomen. Of these, 50 had two episodes, 5 had three, and 2 had four. Figure 1 shows the number and frequency of various acute abdomens. The most common diseases were acute pancreatitis (4.0%), ileus (1.2%), and acute appendicitis (0.7%).

### 3.2 Risk Factors

The basic characteristics of patients with and without acute abdomen are listed in Table 1. There was no significant difference in acute abdomen incidence between males and females (6.6% vs. 6.9%, *p* = 0.64). The incidence of acute abdomen was related to age, with the patients over 10 years old higher than those under 1 year old and between 1 and 10 years old (13.0% vs. 5.7%, *p* < 0.001). Among all types, differences varied with age in acute pancreatitis, acute appendicitis, enterorrhagia, and peritonitis were statistically significant (Table S1). The incidence of acute abdomen was higher in newly diagnosed patients with a peripheral blood leukocyte count ≥ 50 × 10^9^/L than in those with < 50 × 10^9^/L (10.1% vs. 5.8%, *p* < 0.0001), with significant differences in the incidence of acute pancreatitis, enterorrhagia, and enteritis (Table S2). Patients with T‐ALL had a higher incidence of acute abdomen than those with B‐ALL (12.3% vs. 6.1%, *p* < 0.001), with significant differences in acute pancreatitis and enterorrhagia rates (Table S1). The incidence of acute abdomen varied among patients with different risk stratification, higher in the I/HR group than in the LR group (9.9% vs. 3.7%, *p* < 0.001). Statistically significant differences were observed in the incidences of acute pancreatitis, acute appendicitis, ileus, enterorrhagia, and enteritis between the two groups (Table 2). Patients positive for BCR‐ABL1 had a higher incidence of acute abdomen than those negative for BCR‐ABL1 (17.9% vs. 6.2%, *p* < 0.001), with a higher rate of enterorrhagia (3.0% vs. 0.4%, *p* < 0.001) (Table S2). In acute lymphoblastic leukemia, risk factors such as age, initial white blood cell count, immunophenotyping, and risk stratification, the incidence rates of acute pancreatitis and enterorrhagia showed statistically significant differences.

**TABLE 1:** Characteristics of patients with acute abdomen and without acute abdomen.

Variables	No. of patients	No. (%) of patients with acute abdomen (*n* = 512)	*p*
Gender			0.64
Male	4522	298 (6.6%)	
Female	3118	214 (6.9%)	
Age			< 0.0001
0 < Y < 1	134	6 (4.5%)	
1 ≤ Y < 10	6481	373 (5.8%)	
Y ≥ 10	1025	133 (13.0%)	
WBC			< 0.0001
≥ 50	1611	162 (10.1%)	
< 50	6029	350 (5.8%)	
Immunophenotype			< 0.0001
B	6916	423 (6.1%)	
T	724	89 (12.3%)	
BCR‐ABL1			< 0.0001
Positive	335	60 (17.9%)	
Negative	7305	452 (6.2%)	
Final risk			< 0.0001
LR	3948	147 (3.7%)	
IR	3543	352 (9.9%)	
HR	149	13 (8.7%)	

**TABLE 2:** Distribution of different types of acute abdomen in different risk.

	Low risk (*n* = 3948)	Intermediate/high risk (*n* = 3692)	*p*
No. of acute abdomen	147	365	< 0.0001
No. of acute abdomen episodes	156 (4.0%)	422 (11.4%)	< 0.0001
Acute Pancreatitis (*n* = 346)	91 (2.3%)	255 (6.9%)	< 0.0001
Acute Appendicitis (*n* = 51)	19 (0.5%)	32 (0.9%)	0.048
Ileus (*n* = 96)	30 (0.8%)	66 (1.8%)	< 0.0001
Enterobrosis (*n* = 11)	4 (0.1%)	7 (0.2%)	0.37
Enterorrhagia (*n* = 40)	7 (0.2%)	33 (0.9%)	< 0.0001
Peritonitis (*n* = 6)	1 (0.03%)	5 (0.1%)	0.11
Enteritis (*n* = 24)	3 (0.08%)	21 (0.6%)	0.0001
Others (*n* = 4)	1 (0.03%)	3 (0.08%)	0.36

### 3.3 Treatment Phase of Acute Abdomen

Of 578 acute abdomen episodes, most occurred during the induction phase, accounting for 331 cases (57.3%). The continuation and reinduction stages accounted for 177 cases (30.6%), the maintenance stage 51 cases (8.8%), and the consolidation stage the fewest at 19 cases (3.3%). In the LR group, 95 cases occurred in the phase of induction (60.9%), 44 in the continuation and reinduction stage (28.2%), 14 in the maintenance stage (9.0%), and 3 in the consolidation stage (1.9%). In the I/HR group, 236 cases occurred in the phase of induction (55.9%),133 in the continuation and reinduction stage (31.5%), 37 in the maintenance stage (8.8%), and 16 in the consolidation stage (3.8%) (Table 3). The induction stage saw the highest incidence of common acute abdomen, followed by the continuation and reinduction stage, except for acute appendicitis. The incidence rates of acute pancreatitis, acute appendicitis, ileus, enterobrosis, enterorrhagia, peritonitis, and enteritis showed statistically significant variations across different chemotherapy stages (Table 4).

**TABLE 3:** Distribution of acute abdomen in different risk and treatment phases.

	Low risk (*n* = 3948)	Intermediate/high risk (*n* = 3692)
No. of patients with acute abdomen	147 (3.72%)	365 (9.89%)
No. of acute abdomen episodes	156	422
Induction (weeks 1–7)	95 (60.9%)	236 (55.9%)
Consolidation (weeks 8–15)	3 (1.92%)	16 (3.79%)
Continuation and reinduction (weeks 16–34)	44 (28.2%)	133 (31.5%)
Maintenance (weeks 35–125)	14 (8.97%)	37 (8.77%)

**TABLE 4:** Distribution of different types of acute abdomen in different chemotherapy stages.

Number of acute abdomen episodes	Induction (weeks 1–7)	Consolidation (weeks 8–15)	Continuation and reinduction (weeks 16–34)	Maintenance (weeks 35–125)	*p*
Acute pancreatitis (*n* = 346)	193 (55.8%)	5 (1.4%)	123 (35.5%)	25 (7.2%)	< 0.0001
Acute appendicitis (*n* = 51)	26 (51%)	4 (7.8%)	9 (17.6%)	12 (23.5%)	< 0.0001
Ileus (*n* = 96)	52 (54.1%)	3 (3.1%)	33 (34.4%)	8 (8.3%)	< 0.0001
Enterobrosis (*n* = 11)	10 (90.9%)	0 (0)	1 (9.1%)	0 (0)	< 0.0001
Enterorrhagia (*n* = 40)	27 (67.5%)	3 (7.5%)	6 (15%)	4 (10%)	< 0.0001
Peritonitis (*n* = 6)	4 (66.7%)	1 (16.7%)	1 (16.7%)	0 (0)	0.046
Enteritis (*n* = 24)	18 (75%)	2 (8.3%)	4 (16.7%)	0 (0)	< 0.0001
Others (*n* = 4)	1 (25%)	1 (25%)	0 (0)	2 (50%)	0.44

### 3.4 Treatment and Outcome

During treatment, 22 patients (3.8%) with acute abdomen underwent surgery, including 6 cases of acute appendicitis, 11 of enterobrosis, 2 of ileus, 2 of enterorrhagia, and 1 case of abdominal abscess caused by abdominal infection (Table S3). Among 578 acute abdomen episodes, 90 episodes required transfer to ICU for treatment, and 8 patients were transferred to ICU twice (Table S4). Among all types of acute abdomen, enterobrosis had the highest ICU transfer rate (7/11, 63.6%), followed by enterorrhagia (13/40, 32.5%). Although ileus was common among acute abdomen patients, none were transferred to the ICU for ileus‐related issues. In total, nine patients with acute abdomen discontinued the treatment protocol due to pancreatitis, as decided by their treating physicians. In sum, the difference in protocol discontinuation rates, as decided by treating physicians, between patients with and without acute abdomen was not statistically significant [9/512 (1.8%) vs. 99/7128 (1.4%), *p* = 0.44]. During acute abdomen treatment, 10 patients discontinued treatment: 8 due to severe conditions, 1 due to economic reasons, and 1 due to personal reasons. Patients who discontinued treatment were excluded from the death statistics. Of the remaining patients, 16 (3.2%) died directly from acute abdomen (Table 5). Patients with T‐ALL had a higher fatality rate from acute abdomen than those with B‐ALL [6/87 (6.9%) vs. 10/415 (2.4%), *p* = 0.04]. The incidence of death due to acute abdomen in patients who were transferred to ICU was higher than those not transferred to ICU [11/72 (15.3%) vs. 5/430 (1.2%), *p* < 0.0001]. Among acute abdomen, enterorrhagia had the highest mortality rate (6/36, 16.7%), exceeding all other types combined (16.7% vs. 2.15%, *p* < 0.001).

**TABLE 5:** Characteristics of the number of deaths directly caused by acute abdomen.

Variables	No. of acute abdomen (*n* = 502)	No. of deaths (*n* = 16)	*p*	OR
Gender				
Male	292	11 (3.8%)	0.45	1.6
Female	210	5		
Age				
Y < 10	374	10 (2.7%)	0.26	0.56
Y ≥ 10	128	6		
WBC				
< 50	344	12 (3.5%)	0.79	1.39
≥ 50	158	4		
Immunophenotype				
T‐ALL	87	6	0.04	3.0
B‐ALL	415	10 (2.4%)		
Final risk				
LR	144	4	> 0.99	0.82
I/HR	358	12 (3.4%)		
ICU				
Yes	72	11 (15.3%)	< 0.0001	15.3
No	430	5		
Surgical operation				
Yes	21	1	0.51	1.6
No	481	15 (3.3%)		
Enterorrhagia				
Yes	36	6 (16.7%)	0.0004	9.12
No	466	10 (2.1%)		

Abbreviation: OR, odds ratio.

## 4 Discussion

Acute lymphoblastic leukemia is the most common malignancy in the pediatric population, with an incidence of approximately 44.8 per million [5]. As understanding of its clinical, immunological, genetic, and molecular features deepens, risk‐stratified treatment plans have been refined, with long‐term EFS progressively increasing to 70%–85% [7, 8, 9, 10]. The 5‐year cumulative risk of death during remission was 1.3%, outperforming other reports [5]. With continuous improvements in the prevention and management of associated complications, treatment‐related mortality has decreased compared with earlier levels. However, treatment‐related complications remain an important factor affecting the prognosis and long‐term quality of life for ALL patients [11]. Acute abdomen is a frequent treatment‐related complication during acute lymphoblastic leukemia therapy. Studies show that among hematologic malignancy patients, 1.9% to 2.3% have acute abdomen [12], while this rate is higher at 5.3% for acute leukemia patients [13]. The 6.7% incidence rate of acute abdomen among ALL patients in this study is consistent with prior reports. Among the types of acute abdomen statistically analyzed in this study, acute pancreatitis is the most common, followed by ileus, acute appendicitis, and enterorrhagia. The incidence of acute appendicitis in this study was 0.7%, which is similar to the incidence of acute appendicitis during the treatment of hematological diseases in the local cancer center reported in Hannah von Mersi et al., which was 0.9% [14]. The incidence of appendicitis in hematologic malignant patients reported in several previous studies ranged from 0.3% to 1.5% [15, 16, 17].

Notably, over half of these cases occurred during the induction remission stage. During this stage, patients' immunity system is severely compromised, due to extensive leukemia cell infiltration and severe bone marrow suppression from high‐intensity chemotherapy's toxic effects. Consistently, a single‐center Chinese study of 734 ALL children showed severe adverse events were predominantly observed during the induction chemotherapy phase [18]. Similarly, in Sofie et al.'s study involving 313 children with acute myeloid leukemia, abdominal complications mainly occurred during induction chemotherapy [19]. During this phase, prolonged steroid therapy and intensive combination chemotherapy induce severe bone marrow suppression. All these result in atypical presentations or asymptomatic cases of acute abdomen, complicating early clinical diagnosis and treatment [20, 21, 22]. Better use of laboratory and image‐assisted examinations is conducive to early identification of acute abdomen [6, 23]. Therefore, induction stage is a crucial phase in the prevention and treatment of acute abdominal conditions and other adverse reactions, necessitating intensive nursing care and comprehensive supportive therapy.

During the treatment of ALL patients, chemotherapy intensity varies according to different risk stratification. High white blood cell count, age over 10 years, poor genetic subtypes, and high minimal residual disease are all risk factors for ALL. Patients in the I/HR group receive more intensive chemotherapy than those in the LR group leading to prolonged myelosuppression and immunosuppression, consequently increasing the risk of treatment‐related complications. A retrospective study found neutropenic enteropathy, an abdominal complication, most occurred in the I/HR group during childhood leukemia treatment [24]. Our previous findings indicated that the ALL I/HR group exhibited a significantly higher incidence of severe adverse reactions, particularly sepsis, compared to the LR group [25]. The incidence of acute abdomen in patients ≥ 10 years old was higher than those 1–10 years old, with acute pancreatitis having the highest incidence rate. Supportively, a systematic review found that patients ≥ 10 years old have more than twice the risk of asparaginase‐related acute pancreatitis compared to younger patients, with a higher incidence in the HR group [26, 27, 28].

In this study, 16 patients died directly from acute abdomen, mainly in the I/HR group, with a mortality rate of 3.2%. Immunophenotyping, ICU admission, and enterorrhagia are all independent risk factors for mortality. Among all types of acute abdomen, enterorrhagia has the highest mortality rate (16.7%) and predominantly occurs during the induction remission phase. Hematopoietic inhibition early in chemotherapy, vascular endothelial cell damage from tumor cell infiltration, and asparaginase‐associated coagulopathy [18, 29] are all high‐risk factors for bleeding during the induction remission phase. In our study, enterorrhagia incidence was higher in patients < 1 year and ≥ 10 years old than in those 1–10 years old. BCR‐ABL1‐positive children with acute abdomen had a higher enterorrhagia incidence than BCR‐ABL1‐negative patients. This may be related to the use of targeted tyrosine kinase inhibitors with chemotherapy and their associated adverse effects in BCR‐ABL1‐positive patients [30].

The role of immunotherapy in B‐ALL is now well‐established [31]. Blinatumomab, a bispecific T‐cell engager (BiTE) immunotherapy targeting CD19 on B‐cells and CD3 on T‐cells, has revolutionized the treatment of pediatric B‐ALL. Initially approved for relapsed/refractory (R/R) disease, its use is now expanding into frontline therapy, particularly for high‐risk patients, offering a targeted approach with reduced toxicity compared to intensive chemotherapy [32]. The result from the AIEOP‐BFM ALL 2017 study further demonstrated the toxicity profile of Blinatumomab is much more favorable as compared to the intensive chemotherapy approach [33]. Thus, the introduction of immunotherapy in B‐ALL will mitigate the adverse effects associated with intensified chemotherapy, representing an inevitable trend for the future. Most literature reports that infection is the main cause of serious adverse reactions in the treatment of hematologic malignancies [11, 18, 25]. Patients suffering from concurrent abdominal complications and sepsis had a high risk of treatment‐related mortality [19]. This study did not further analyze the occurrence and possible risk factors of infection‐related and non‐infection‐related acute abdomen. Surgical treatment is a crucial modality in the management of acute abdomen. Many domestic and foreign studies have indicated that neutropenia in patients with hematologic malignancies is not a contraindication to surgery [15, 16, 17]. Appropriate surgical timing and sufficient support can decrease mortality [3, 14]. This study lacks relevant analysis in this aspect and requires further improvement in the future.

In summary, this study analyzed the incidence and related risk factors of acute abdomens in the CCCG‐ALL‐2015 protocol, with an overall incidence of 6.7%. The incidence of different types of acute abdomen is associated with the treatment phase, risk stratification, and age. Acute abdomen had the highest incidence during the induction phase, and the incidence in the IR/HR group was higher than in the LR group. The incidence of acute pancreatitis and enterorrhagia varied across different age groups. In this study, acute pancreatitis had the highest incidence among acute abdomens, while enterorrhagia had the highest mortality rate.

## Author Contributions

**Wenting Gan:** writing – original draft, formal analysis. **Weina Zhang:** writing – review and editing, formal analysis. **Jiaoyang Cai:** writing – review and editing, data curation. **Xiaofan Zhu:** resources. **Jie Yu:** resources. **Peifang Xiao:** resources. **Ju Gao:** resources. **Yongjun Fang:** resources. **Changda Liang:** resources. **Xue Li:** resources. **Fen Zhou:** resources. **Xiaowen Zhai:** resources. **Xiaoxiao Xu:** resources. **Xin Tian:** resources. **Aiguo Liu:** resources. **Ningling Wang:** resources. **Jiashi Zhu:** resources. **Frankie Wai‐Tsoi Cheng:** resources. **Liangchun Yang:** resources. **Ge Zhang:** resources. **Shuhong Shen:** resources. **Hua Jiang:** writing – review and editing, supervision, methodology. **Lingzhen Wang:** writing – review and editing, supervision.

## Ethics Statement

This multicenter study was approved by the Ethical Committee of Guangzhou Women and Children's Medical Center (Approval no. 2015020936) and the local ethics committees of all participating centers. We obtained written informed consent from the parents or guardians of all the patients.

## Conflicts of Interest

The authors declare no conflicts of interest.

## Supporting information


Data S1.

## Data Availability

The data are available from the corresponding author upon reasonable request.
