# Supplementary material for: A Multicenter Study of Acute Abdomen in Children With Acute Lymphoblastic Leukemia: CCCG‐ALL‐2015
Source: Cancer Med. 2025 Aug 21;14(16):e71090. doi: 10.1002/cam4.71090 (PMC12370538; doi:10.1002/cam4.71090)
Supplement: Supplementary file 1 — Data S1. [file CAM4-14-e71090-s001.docx]

**Supplementary TABLE 1** Different types of acute abdomen distributed in different age, WBC, and immunophenotype groups

|  | **0<Y<1**  **(n=134)** | **1<=Y<10**  **(n=6481)** | **Y≥10**  **(n=1025)** | ***P* value** | **WBC≥50**  **(n=1611)** | **WBC<50**  **(n=6029)** | ***P* value** | 1. **ALL**   **(n=6916)** | 1. **ALL**   **(n=724)** | ***P* value** |
| --- | --- | --- | --- | --- | --- | --- | --- | --- | --- | --- |
| **No. of acute abdomen** | 6 | 373 | 133 | <0.0001 | 162 | 350 | <0.0001 | 423 | 89 | <0.0001 |
| **No. of acute abdomen episodes** | 6(4.5%) | 418(6.4%) | 154(13%) | <0.0001 | 187(11.6%) | 391(6.5%) | <0.0001 | 476(6.9%) | 102(14%) | <0.0001 |
| **Acute Pancreatitis**  **(n=346)** | 1(0.7%) | 243(3.7%) | 102(10%) | <0.0001 | 108(6.7%) | 238(3.9%) | <0.0001 | 279(4.0%) | 67(9.2%) | <0.0001 |
| **Acute Appendicitis**  **(n=51)** | 0(0%) | 38(0.6%) | 13(1.3%) | 0.028 | 13(0.8%) | 38(0.6%) | 0.49 | 42(0.6%) | 9(1.2%) | 0.054 |
| **Ileus(n=96)** | 1(0.7%) | 83(1.3%) | 12(1.2%) | 0.83 | 27(1.7%) | 69(1.1%) | 0.1 | 85(1.2%) | 11(1.5%) | 0.48 |
| **Enterobrosis(n=11)** | 0(0%) | 8(0.1%) | 3(0.3%) | 0.37 | 5(0.3%) | 6(0.1%) | 0.06 | 9(0.1%) | 2(0.3%) | 0.28 |
| **Enterorrhagid(n=40)** | 3(2.2%) | 24(0.4%) | 13(1.3%) | <0.0001 | 23(1.4%) | 17(0.3%) | <0.0001 | 32(0.5%) | 8(1.1%) | 0.049 |
| **Peritonitis(n=6)** | 0(0%) | 2(0.03%) | 4(0.4%) | 0.0007 | 0(0%) | 6(0.1%) | 0.35 | 6(0.09%) | 0(0%) | >0.99 |
| **Enteritis(n=24)** | 1(0.7%) | 17(0.3%) | 6(0.6%) | 0.15 | 10(0.6%) | 14(0.2%) | 0.02 | 20(0.3%) | 4(0.6%) | 0.28 |
| **Other(n=4)** | 0(0%) | 3(0.05%) | 1(0.1%) | 0.77 | 1(0.06%) | 3(0.05%) | >0.99 | 3(0.04%) | 1(0.1%) | 0.33 |

**Supplementary TABLE 2** Comparison of the incidence of enterorrhagia in BCR-ABL1 positive and BCR-ABL1 negative patients

| **BCR-ABL1 fusion gene** | Enterorrhagia | Without Enterorrhagia | Total | *P* value |
| --- | --- | --- | --- | --- |
| **Positive** | 10 | 325 | 335 | <0.0001 |
| **Negative** | 29 | 7276 | 7305 |  |
| **Total** | 39 | 7601 | 7640 |  |

**Supplementary TABLE 3** Number of patients undergoing surgical operations during treatment of acute abdomen

| **No. of acute abdomen episodes** | **Surgical operation(n=22)** | |
| --- | --- | --- |
| Acute Pancreatitis (n=346) | | 0 (0%) |
| Acute Appendicitis (n=51) | | 6 (11.8%) |
| Ileus (n=96) | | 2 (2.1%) |
| Enterobrosis (n=11) | | 11 (100%) |
| Enterorrhagia (n=40) | | 2 (5.0%) |
| Peritonitis (n=6) | | 1 (16.7%) |
| Enteritis (n=24) | | 0 (0%) |
| Others (n=4) | | 0 (0%) |

**Supplementary TABLE 4** Number of ICU transfers during the treatment of acute abdomen

| **Variables** | **ICU(n=79)** |
| --- | --- |
| Acute Pancreatitis (n=346) | 64(18.5%) |
| Acute Appendicitis (n=51) | 1(2.0%) |
| Ileus (n=96) | 0(0%) |
| Enterobrosis (n=11) | 7(63.6%) |
| Enterorrhagia (n=40) | 13(32.5%) |
| Peritonitis(n=6) | 1(16.7%) |
| Enteritis (n=24) | 4(16.7%) |
| Others (n=4) | 0(0%) |
